# *Skeletonema marinoi* as a new genetic model for marine chain-forming diatoms

**DOI:** 10.1038/s41598-019-41085-5

**Published:** 2019-04-02

**Authors:** Oskar N. Johansson, Mats Töpel, Matthew I. M. Pinder, Olga Kourtchenko, Anders Blomberg, Anna Godhe, Adrian K. Clarke

**Affiliations:** 1 Department of Biological and Environmental Sciences, University of Gothenburg, Box 461, SE-40530 Gothenburg, Sweden; 2 Department of Marine Sciences, University of Gothenburg, Box 462, SE-40530 Gothenburg, Sweden; 3 Gothenburg Global Biodiversity Center (GGBC), Box 461, SE-40530 Gothenburg, Sweden; 4 Department of Chemistry and Molecular Biology, University of Gothenburg, Box 462, SE-40530 Gothenburg, Sweden

## Abstract

Diatoms are ubiquitous primary producers in marine ecosystems and freshwater habitats. Due to their complex evolutionary history, much remains unknown about the specific gene functions in diatoms that underlie their broad ecological success. In this study, we have genetically transformed the centric diatom *Skeletonema marinoi*, a dominant phytoplankton species in temperate coastal regions. Transformation of *S*. *marinoi* is the first for a true chain-forming diatom, with the random genomic integration *via* nonhomologous recombination of a linear DNA construct expressing the resistance gene to the antibiotic zeocin. A set of molecular tools were developed for reliably identifying the genomic insertion site within each transformant, many of which disrupt recognizable genes and constitute null or knock-down mutations. We now propose *S*. *marinoi* as a new genetic model for marine diatoms, representing true chain-forming species that play a central role in global photosynthetic carbon sequestration and the biogeochemical cycling of silicates and various nutrients, as well as having potential biotechnological applications.

## Introduction

Phytoplankton are the foundation of the marine food web that sustains most oceanic life. Diatoms are distinguished from other phytoplankton by their elaborate cell wall, or frustule, composed of biogenic silicates organized in a myriad of intricate shapes and patterns^1^. They are the most diverse of the photosynthetic eukaryotic lineage and are responsible for ca. 20% of global photosynthetic activity^2^. Those diatoms forming long chains are especially important to the global biogeochemical cycles by sequestration and mineralization of carbon, nitrogen and silica. They form fast-sinking aggregates that over time bury fixed atmospheric carbon dioxide in ocean floor sediments, thereby being responsible for much of the oceanic gas and oil reserves. Chain-forming diatoms are also dominant primary producers in spring blooms in the polar oceans and temperate regions, as well as in subtropical and tropical upwelling regions where they support fish production. One of the most common genera of chain-forming diatoms is *Skeletonema* that dominates most coastal waters^3^. In Scandinavian waters, the species *S*. *marinoi* is a crucial primary producer throughout the year, especially during spring blooms. It also forms benthic resting spores that can survive in sediments for more than a century, facilitating micro-evolutionary studies of temporal changes in diatom ecology and genetics^4,5^.

Several theories have been proposed for the still-inexplicable global success of chain-forming diatoms, and how chain formation may have provided adaptations to the physical, chemical, and biological constraints of marine life. Equally uncertain is the extent to which this prominent group of phytoplankton will be impacted in the future by increasing environmental pressures from anthropogenically-driven climate change. The ecological success of diatoms is influenced by their complex evolutionary history that includes multiple rounds of endosymbiosis and horizontal gene transfer, resulting in an unusual gene composition^6^. This is exemplified by the existence of genes in diatom genomes for a metazoan-like ornithine/urea cycle that is absent in plants and other types of algae^7^. The nuclear genomes of several different diatoms have now been sequenced^8–12^, including the pennate *Phaeodactylum tricornutum* and centric *Thalassiosira pseudonana*. These sequences have revealed a large proportion of hypothetical proteins of unknown function (ca. 40%), as well as many with only marginal similarity to known proteins in other organisms^8,12,13^. Even for those genes encoding identifiable proteins, the extent of their functions and overall importance within the cellular regulatory networks in different diatoms are far from clear. As a consequence, making precise gene function determinations for diatoms based on studies of other photosynthetic organisms is considerably less informative and potentially misleading.

We have recently sequenced the nuclear genome of the pelagic, bipolar, centric diatom *S*. *marinoi* (http://cemeb.science.gu.se/research/target-species-imago%20/skeletonema*- marinoi*, Töpel *et al*. in prep.), which is a prominent phytoplankton species in temperate coastal waters^14^. The completed genome sequence of *S*. *marinoi* is the first for a true chain-forming diatom species, and as such is representative of those diatoms most influential in the marine carbon cycle and biogeochemical activities. The current annotation of the *S*. *marinoi* genome reveals 22,440 genes, of which 79% encode proteins of unknown function. Historically, the most successful genetic strategy for elucidating the specific function of these numerous hypothetical proteins in many different organisms has been mutagenesis using either forward or reverse genetics. In this regard, large collections of random insertional mutants suitable for both single gene studies and large-scale phenotyping have been of particular value as exemplified by the vast volume of knowledge gained over many years from the T-DNA mutant collections for the vascular plant *Arabidopsis thaliana*^15^. Despite the tremendous success of such mutant collections, no such approach has yet been applied to a diatom genome, with single-gene mutagenesis studies limited to only a few diatom species so far.

Central to the suitability of a species to such mutagenesis studies is its capacity for genetic transformation. In this study, we successfully transformed the first centric, chain-forming diatom, *S*. *marinoi*, taking advantage of the characteristic shared by most protists of randomly integrating exogenous DNA within the nuclear genome *via* nonhomologous recombination. One important distinction was the relatively sensitivity of *S*. *marinoi* to antibiotic selection compared to that for transformations of other diatoms, most of which were with pennate species. PCR-based approaches were then developed to map for the first time in a diatom the genomic insertion site within a transformed line. As proof-of-concept of the suitability of *S*. *marinoi* for future mutagenesis studies, the insertion site in selected transformants were mapped, in which most occurred within recognizable gene structures. Of these gene disruptions, ca. one-third were also shown to be homozygous for the mutation, with the others being heterozygous.

## Methods

### Culture conditions

The *S*. *marinoi* isolate R05AC was originally established from a germinated resting stage in 2010 from sediments in Öresund, Sweden (55°59.16 = N, 12°44.02 = E)^16^, while the *P*. *tricornutum* isolate UTEX 642 was obtained from the UTEX Culture Collection of Algae at the University of Texas, USA. For this study, *S*. *marinoi* R05AC was obtained from the Gothenburg University algae bank and maintained in enriched f/2 (with Si addition and filtered, autoclaved sea water at 26 PSU) cultured in 30 ml TC flasks (Sarstedt, Germany) fitted with sterile filters. *P*. *tricornutum* was maintained in the enriched f/2 described above. The standard growth conditions for both diatom species were 16 °C with a 16 h photoperiod at an irradiance of 70 μmol photons m^−2^ s^−1^.

### Antibiotic sensitivity assay

Pre-cultures of *P*. *tricornutum* and *S*. *marinoi* grown under standard conditions in enriched f/2 media to mid-exponential growth phase were used to inoculate experimental cultures in fresh enriched f/2 with different concentrations of zeocin (InVivoGen, USA) in 48-well plates (Corning, USA). Growth was measured daily for one week from chlorophyll *a* fluorescence measurements (425 nm excitation and 680 nm emission wavelengths)^17^ using a plate reader (Varioscan Flash, ThermoScientific, USA).

### DNA constructs for *S*. *marinoi* transformation

Sequences for the *P*. *tricornutum fcpB* promoter/terminator regions and the bleomycin/zeocin resistance gene were obtained from the National Center for Biotechnology Information (NCBI). Sequences for the *lsu4e* promoter/terminator regions were taken from the *S*. *marinoi* reference genome assembly (Töpel *et al*. in prep.) and chosen for their relatively high expression levels in transcriptomics data (data not shown). All non-native sequences were codon optimized for *S*. *marinoi* based on the reference genome assembly and predicted gene models. Both DNA constructs (*fcpB:bleoR:fcpB* and *lsu4e:bleoR:lsu4e*, supplementary Fig. 1) were commercially synthesized (ThermoScientific, USA).

### Genetic transformation of *S*. *marinoi*

Large cultures (800 mL) of *S*. *marinoi* R05AC were grown to mid-exponential growth phase and then harvested by centrifugation (1200 × *g*, 5 min, 4 °C). Cell pellets were resuspended in 10 mL 0.3 M sorbitol, re-centrifuged (1200 × *g*, 5 min, 4 °C) and resuspended again in 0.3 M sorbitol (5 mL). Cell density was determined by chlorophyll *a* fluorescence and adjusted to 5 × 10^7^–2 × 10^8^ cells mL^−1^ depending on the electroporation conditions being tested. Cells were maintained on ice until use. DNA was prepared by PCR amplification from vector constructs (1.5 ng) in 50 μL reactions using 1U Platinum Super Fi DNA polymerase (ThermoScientific, USA) according to the manufacturer’s instructions and a concentration of 0.3 μM for fcpB or Lsu4e primers (Supplementary Fig. 4). Reactions were purified using Wizard PCR purification kit (Promega, USA). Prior to electroporation, cells were manually shaken for 1–2 min to minimize chain length to an average of 2–3 cells. Construct DNA (10 µg) was then added to 100 μL of cell suspension in a pre-chilled (4 °C) electroporation cuvette (Φ = 0.2 mm, Bio-Rad, USA), incubated for one min and then electroporated using the multi-pulse Gene Pulser Xcell electroporator (Bio-Rad, USA). The different electroporation settings tested are indicated in supplementary Figure 3. Following electroporation, cells were transferred into fresh growth media (21 °C) and grown for 2 d under standard conditions. Zeocin (final concentration of 0.15 µg mL^−1^) was later added and the cultures were left for another 2 d. Cells were then harvested by centrifugation (1200 × *g*, 5 min, 4 °C) and resuspended in 1 mL growth media supplemented with 0.15 µg mL^−1^ zeocin. Cells (500 µL suspension) were plated on large petri plates (enriched f/2, 0.9% agar, 0.15 µg mL^−1^ zeocin), with colonies appearing after 21–28 d. Colonies were then transferred to 48-well plates containing 900 µL growth media supplemented with zeocin (0.15 µg mL^−1^). Growth was monitored by chlorophyll fluorescence for up to two weeks, where after true transformants were transferred into the mutant collection. For continuous maintenance of the collection, a microwell plate system with inoculation into fresh media with selection every 10–14 days was established.

### DNA extractions

DNA extractions were performed on *S*. *marinoi* cultures (150 mL) grown under standard conditions for 10–14 d. DNA was extracted by grinding pelleted cells in N_2(L)_ and transferring the powder to pre-chilled microcentrifuge tubes. Plant DNAzol (ThermoScientific, USA) supplemented with RNaseA (100 µg mL^−1^) was then added and DNA extracted according to the manufacturer’s instruction, with the following exception that three ethanol (75%) wash steps were performed instead of one. DNA integrity was assessed after separation by agarose gel electrophoresis and visualized by GelStar staining (Lonza, Switzerland).

### Standard PCR

Unless stated otherwise, standard PCR amplification of DNA was performed on a S1000 Thermal cycler (Bio-Rad, USA) in 25 µL reactions using 1.5 U Amplitaq 360 polymerase (ThermoScientific, USA). Primers (supplementary Fig. 3) were used at a concentration of 0.4 µM, with 100 ng genomic DNA as template. Annealing temperature was 65 °C with 35 cycles performed.

### TAIL-PCR

All thermal asymmetric interlaced-PCR (TAIL-PCR) reactions were performed using a S1000 thermal cycler (Bio-Rad, USA). In the primary PCR reactions (25 µL), 4 µM of each degenerate SAD primer and 0.5 µM of the specific BLEO primers (Promoter/BLEO6, Terminator/BLEO8) were used with extracted genomic DNA (100 ng) from each transformant as template. A diluted aliquot of the primary reaction was then used as a template for the secondary PCR reaction (25 µL) to give a final dilution of 1000 fold. In the secondary reaction, the SAD primers were exchanged for the SSMP primer (1 µM) and promoter/terminator BLEO primers were exchanged to BLEO5 (0.5 µM) and BLEO7 (0.5 µM), respectively. For the tertiary PCR reaction (50 µL), an aliquot of the secondary PCR reaction was used as template at a final dilution of 250-fold. SSMP primer and the third set of nested primers (BLEO3, promoter; BLEO4, terminator) were kept at the same concentration (0.5 µM) for the tertiary reaction. All TAIL-PCR reactions were performed using the TaKaRa rTAQ polymerase (Clontech, USA), with 0.75 U used in the primary and secondary reactions and 1.5 U in the tertiary reaction. The thermal cycling conditions for each step are detailed in supplementary Figure 5. The final DNA products amplified in the tertiary reactions were analyzed by agarose gel electrophoresis and visualized by GelStar staining (Lonza, Switzerland). Selected DNA products were then excised from the agarose gel and purified using the Wizard PCR purification kit (Promega, USA). Purified DNA products were commercially sequenced (Eurofins Genomics, Germany) using either the BLEO3 (promoter side) or BLEO4 (terminator side) primers.

## Results

### Genetic transformation of *Skeletonema marinoi*

**Figure 1:**
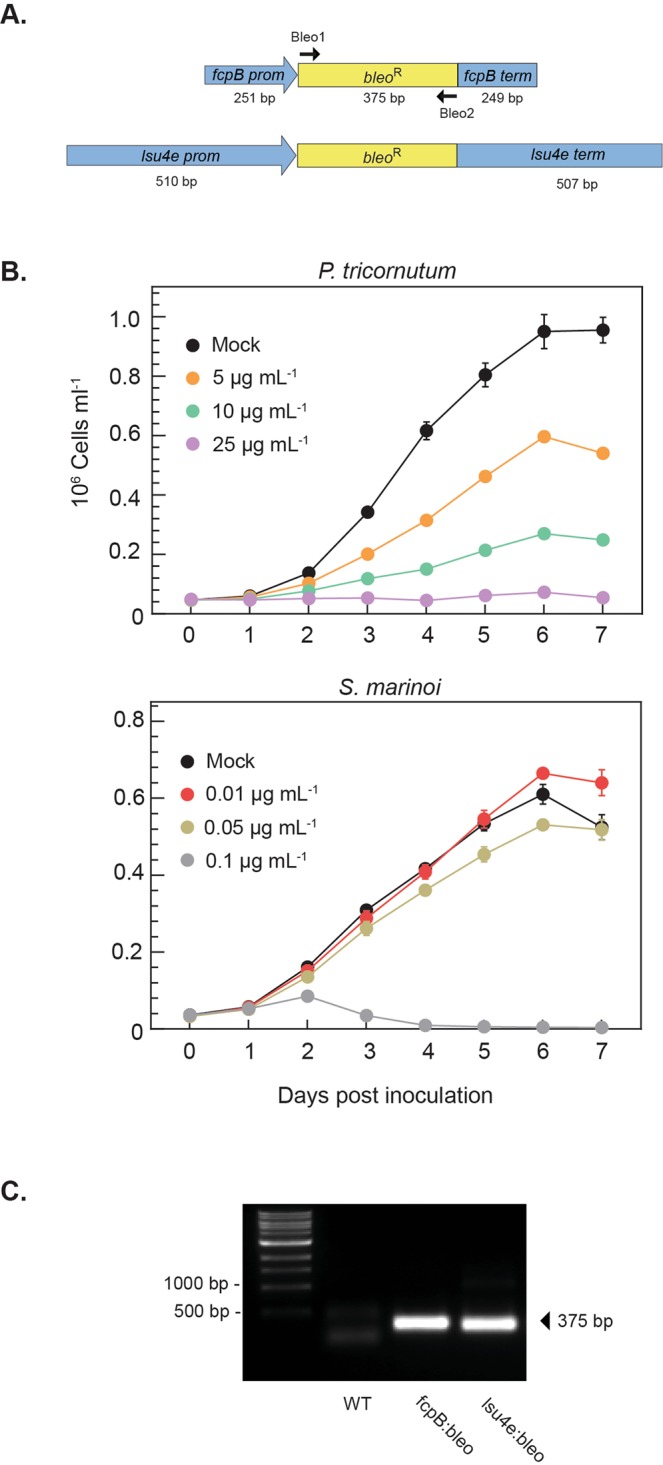
Genetic transformation of *S*. *marinoi*. (**A**) Schematic designs of the linear DNA constructs for the transformation of *S*. *marinoi* R05AC. Two different promoter/terminator regions were chosen to express the selectable marker gene (*bleo*^R^) conferring resistance to the antibiotic zeocin; *fcpB*, gene for the *P*. *tricornutum* fucoxanthin-chlorophyll protein; *lsu4e*, gene for the *S*. *marinoi* 80 S ribosomal large subunit protein 4e. Also shown are the two oligonucleotides (Bleo1 and –2) used to PCR amplify the *bleo*^R^ gene. (**B**) Sensitivity of *S*. *marinoi* to the antibiotic zeocin relative to that of *P*. *tricornutum*. Growth was monitored daily by chlorophyll fluorescence for one week in the presence of different zeocin concentrations. Shown are averages and standard deviation of four replicate cultures. (**C**) Amplification of the *bleo*^R^ gene with Bleo1 and Bleo2 primers in representative transformants using the *fcpB:bleoR* or *lsu4e:bleoR* constructs.

Genetic transformation of diatoms has been shown for several pennate species using mainly microparticle bombardment^18–24^ but also electroporation^25,26^ to deliver relatively long, linearized plasmid DNA that randomly insert into the nuclear genome by nonhomologous recombination. The most common selectable marker included in these constructs is the gene conferring resistance to the antibiotic zeocin (*bleo*^R^). Fewer examples exist for transformation of centric diatoms and none for true chain-forming species such as *S*. *marinoi*. To develop a transformation system for *S*. *marinoi*, we designed minimal-length linear DNA constructs consisting of only the *bleo*^R^ gene regulated by two different promoter/terminator regions, one from the *P*. *tricornutum fcpB* gene and another from the *S*. *marinoi lsu4e* gene that encodes the 80 S ribosomal subunit 4e (Fig. 1A, Supplementary Fig. 1). Prior to transformation, the sensitivity of *S*. *marinoi* to zeocin was determined to choose a suitable concentration for selection of positive transformants. Included for comparison was *P*. *tricornutum*, whose growth was increasingly inhibited at zeocin concentrations up to 25 µg ml^−1^ (Fig. 1B), consistent with selection concentrations of 50–150 µg ml^−1^ used in previous studies^19,27–29^. In contrast, *S*. *marinoi* was unusually sensitive to zeocin concentrations above 0.1 µg ml^−1^, with 0.15 µg ml^−1^ eventually chosen for selection purposes. Although unexpected, the extreme sensitivity of *S*. *marinoi* to this antibiotic is consistent with the failure to produce viable transformants in other centric diatoms selected at higher zeocin concentrations^21,26^.

For *S*. *marinoi* transformation, electroporation using a multi-pulse function with square-wave pore pulses was chosen as the delivery method, with advantages of being relatively inexpensive and less technically demanding than particle bombardment. An overview of the transformation procedure is shown in Supplementary Figure 2. *S*. *marinoi* cultures were taken during exponential growth and briefly shaken prior to electroporation to reduce chain length to an average of two cells. It should be noted that more vigorous or prolonged shaking failed to produce homogeneous single-cell cultures. Various electroporation parameters were then tested for both the poring and transfer pulses, including the voltage, pulse number and length, and the interval between pulses (Supplementary Fig. 3). Following electroporation, cells were grown in liquid media, first without selection for 2 d and then for a further 2 d with zeocin added. Cells were then transferred to selection plates, with single-colony transformants appearing within 3–4 weeks. *S*. *marinoi* transformants were obtained under most of the electroporation conditions tested, with a transformation efficiency similar to that reported for other diatoms^27–29^.

### Identification of the genomic insertion sites

Underpinning the suitability of *S*. *marinoi* as a new genetic model species for future mutagenesis studies is the ability to reliably and precisely identify the genomic insertion site of the transformed DNA construct in each transformant, a procedure that has yet to be described for diatoms. As a proof-of-concept, a subset of the initial transformants was selected for further characterization. Each transformant was transferred from plates to liquid cultures and grown with selection under standard conditions. Total genomic DNA was then extracted and the presence of the construct confirmed in each transformant by PCR amplification using oligonucleotides specific for the *bleo*^R^ gene (Fig. 1A and C).

**Figure 2:**
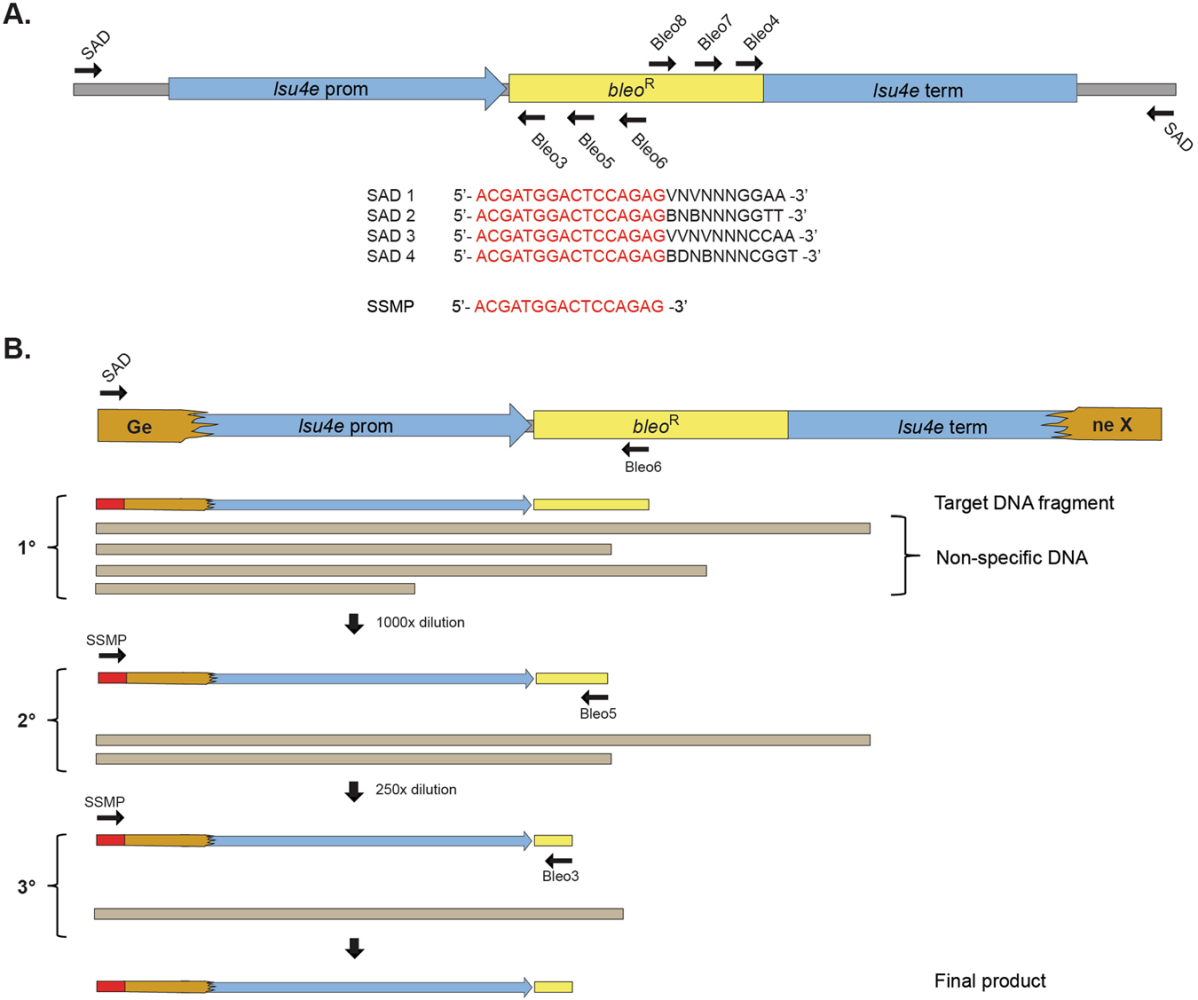
Modified TAIL-PCR approach used to map the genomic insertion sites in *S*. *marinoi* transformants. (**A**) Schematic representation of the two sets of nested oligonucleotides specific for the *bleo*^R^ gene to map the genomic flanking regions at either the promoter (Bleo3, –5, –6) or terminator (Bleo4, -7, -8) end using TAIL-PCR. Each set of nested primers are combined with one of four degenerate primers (SAD1-4) that anneal at a high frequency within the genome. (**B**) Schematic representation of the TAIL-PCR method used to identify the insertion site within an arbitrary gene (gene X) from the promoter end of the construct. In the first round of PCR amplification (1° reaction), the SAD primers produce numerous non-specific products in addition to the target fragment. In the following rounds (2° and 3°), the SAD primer is replaced with the specific SSMP primer, along with additional construct-specific primers (Bleo5 and -3) to concentrate the target fragment. The final products are size separated by gel electrophoresis, with each purified for DNA sequencing. Sequences are then matched against the construct sequence and the R05AC reference genome to identify the insertion site.

**Figure 3:**
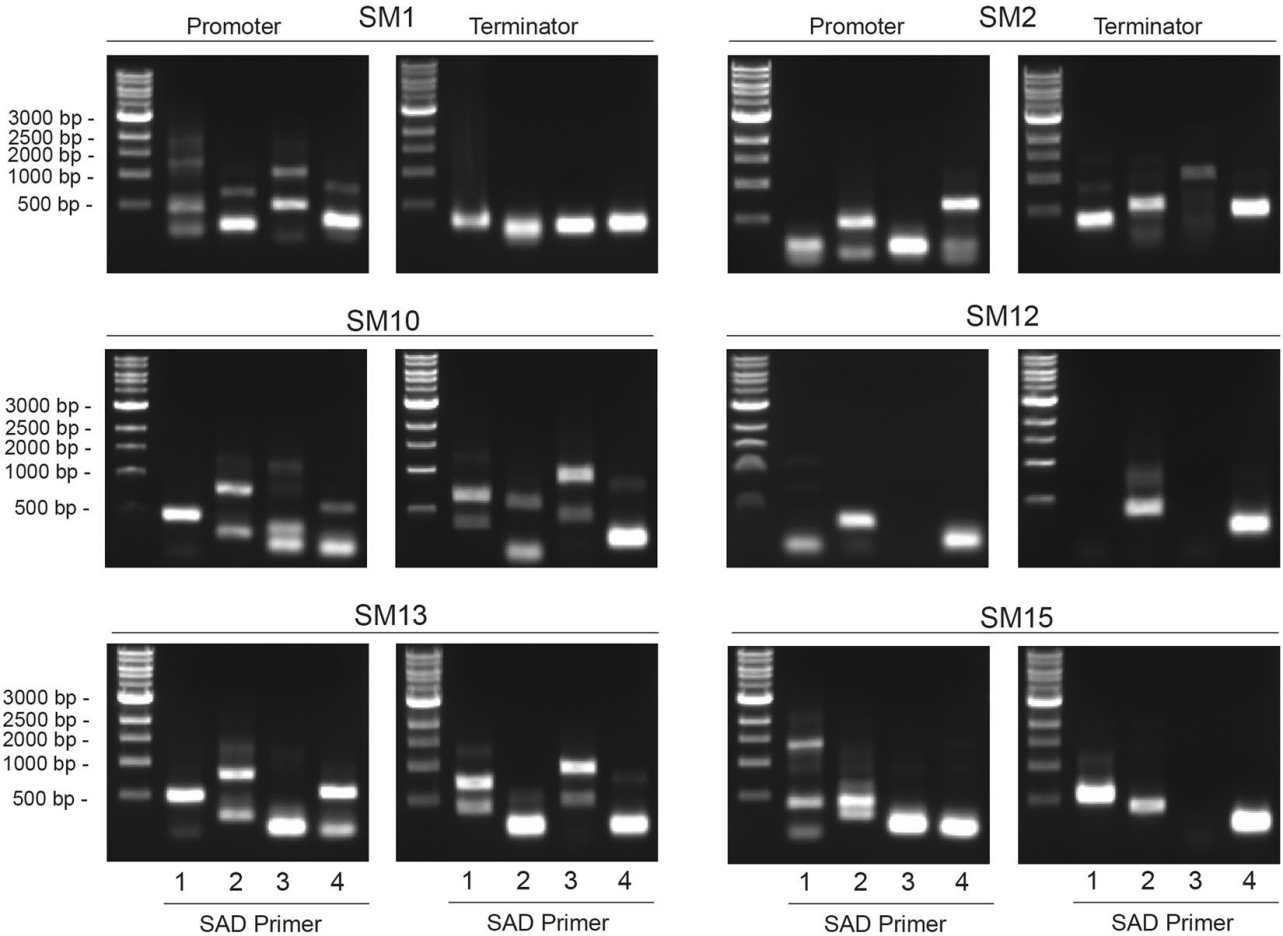
Mapping the genomic insertion sites in several representative transformants using TAIL-PCR. TAIL-PCR was performed on genomic DNA extracted from the transformants SM1–2, SM10, SM12–13 and SM15 using one of the SAD1–4 degenerate primers in each reaction in combination with the construct-specific primers for either the promoter or terminator ends. The final PCR products were separated and visualized by gel electrophoresis as shown, with selected individual products then purified and sequenced.

To identify the genomic insertion site of the construct in each transformant, variations of the TAIL-PCR method were tested on the selected *S*. *marinoi* transformants. Although successful in mapping the T-DNA insertion sites in the vascular plant *A*. *thaliana*^30–32^, none of these published methods worked with *S*. *marinoi* and so a new version was developed (Fig. 2). This TAIL-PCR approach used a combination of nested primers specific for either end of the *bleo*^R^ gene in order to map the insertion site at both ends of the construct, with a set of short arbitrary degenerate primers (SAD, Fig. 2A) that anneal somewhere within the flanking regions (Fig. 2B). The resulting PCR products are then sequenced and the flanking genomic regions identified using the newly available reference genome (Töpel *et al*. in prep.). Of the 16 chosen transformants, 10 were successfully mapped from at least one end of the construct (Fig. 3), whereas the TAIL-PCR produced fragments too short to correctly map the remaining six.

**Figure 4:**
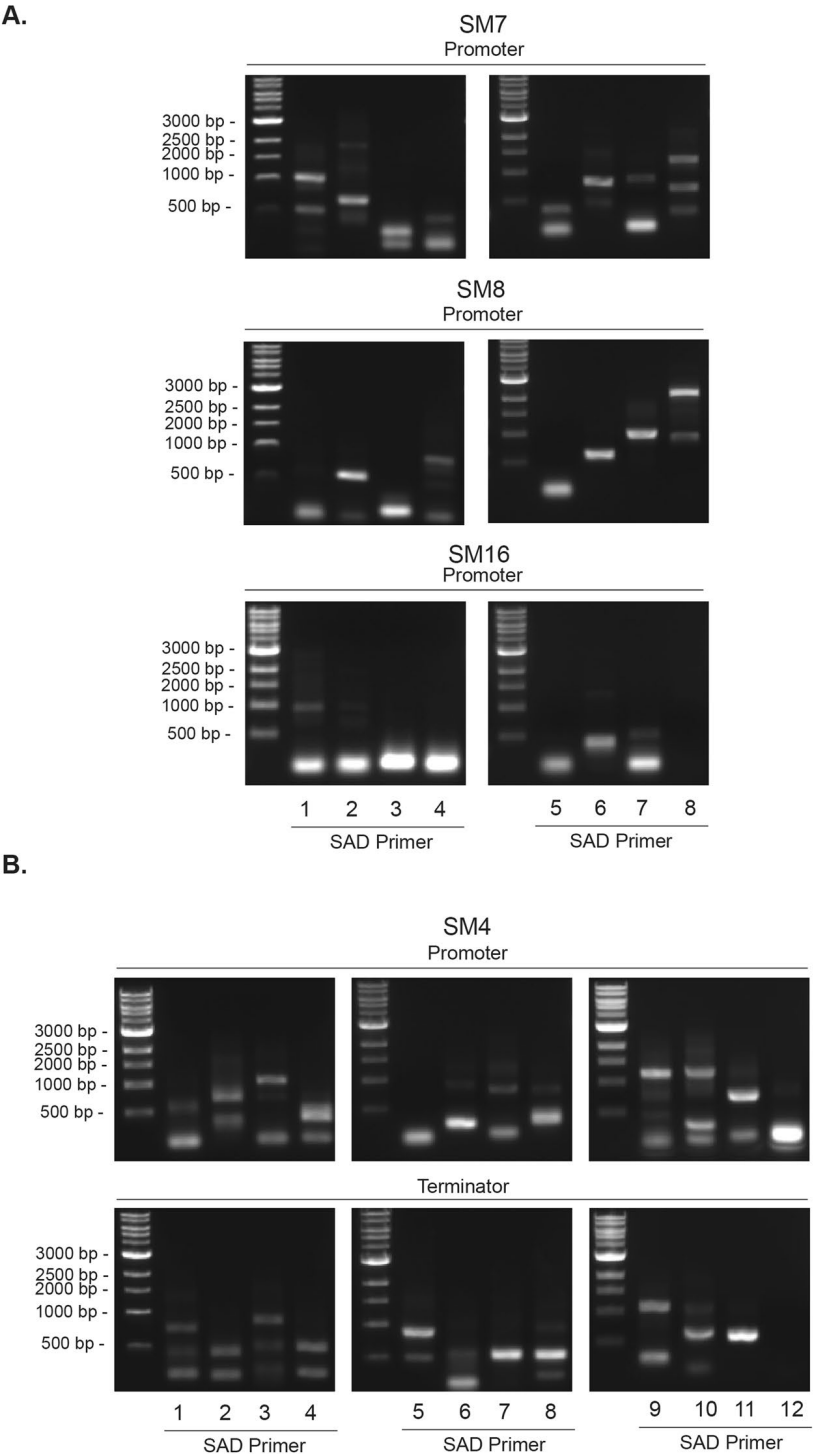
Improved mapping of the genomic insertion sites in selected transformants using TAIL-PCR. Two additional sets of SAD primers (SAD5–8 and SAD9–12) were synthesized in which one or two extra nucleotides, respectively, were added to the 3′ end in order to reduce their annealing frequency within the *S*. *marinoi* genome. Shown are three representative transformants (SM7–8 and SM16) mapped from the promoter end using SAD1–8 (**A**) and one (SM4) mapped from the both ends using SAD1–12 (**B**) in which significantly longer PCR products were produced relative to those using the shorter SAD primers. The final PCR products were separated and visualized by gel electrophoresis, with selected individual products then purified and sequenced.

Due to the inability to identify the genomic insertion site in certain transformants at both ends or not at all because of too short PCR products, we designed another set of SAD primers (SAD5–8) in which an extra nucleotide was added to the 3′ end and the annealing temperature specific for the degenerate primers increased from 25 to 30 °C (Supplementary Fig. 5), thereby reducing the frequency at which the SAD primers anneal to a specific sequence in the genome. For most transformants, the new SAD primers produced considerably longer PCR products (Fig. 4A), the sequencing of which provided a more reliable identification of the insertion site. A third set of SAD primers (SAD9–12, Fig. 4B) were also tested in which another nucleotide was added to the 3′ end of SAD5–8 and their annealing temperature raised to 35 °C to provide an additional option for mapping the genomic insertion sites of transformants for which the first two sets of SAD primers might prove inadequate.

Altogether, the use of the additional SAD primers enabled successful mapping of all but four of the chosen transformants. The insertion in two of these four were mapped to two repeat regions, both of which were near identical in sequence and impossible to distinguish by PCR. The remaining two transformants were mapped but to two different genomic regions from the promoter and terminator ends. This unusual characteristic has been previously described for similar transformations based upon nonhomologous recombination in the green algae *Chlamydomonas reinhardtii*, and believed to be caused by fragmented genomic DNA from cells lysed during electroporation ligating to one end of the DNA construct^33^. Despite the small sample size, the occurrence of such chimeric insertions in *S*. *marinoi* appears less frequent than in *C*. *reinhardtii*, which has been estimated to ca. 40% of transformants^34^, but it is a characteristic that must be continuously monitored once more transformants are mapped.

**Figure 5:**
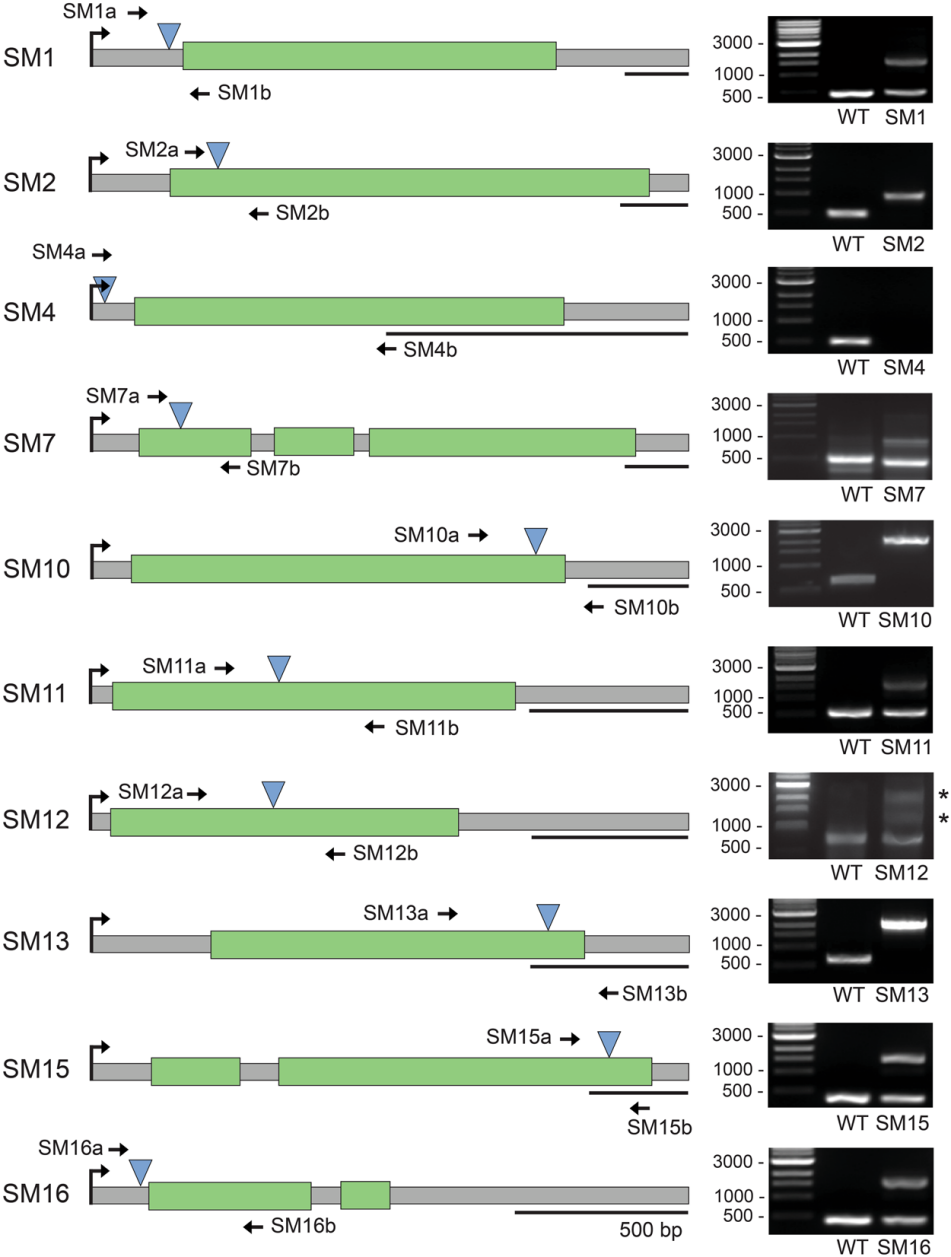
Schematic representation of the genomic insertion site in selected *S*. *marinoi* transformants. Shown on the left are the predicted gene models disrupted by the insertion of the transformed DNA construct (blue triangle) in ten transformants (SM1–2, SM4, SM7, SM10–13 and SM15–16), with the green regions representing exons. The shown genomic regions correspond to at least one mRNA identified by RNA sequencing, the 5′ end of which is indicated by the black arrow. Underneath each gene map is shown a scale corresponding to 500 bp. Also indicated for each transformant are the primers specific for regions flanking the inserted construct used to determine the segregation state as shown on the right, confirming either heterozygosity or homozygosity for the mutation. PCR was performed on genomic DNA extracted from wild type *S*. *marinoi* and the indicated transformant. The amplified products were separated and visualized by gel electrophoresis. The identity of all PCR products was confirmed by DNA sequencing, with non-specific products denoted with an asterisk.

Genomic mapping of the first transformants also revealed that the inserted DNA construct was often truncated to varying extents at either or both the promoter and terminator regions. This phenomenon, which again was also reported in transformation studies of *C*. *reinhardtii*^33^ could explain why more transformants were obtained with the *lsu4e* constructs. Since minimal sequences for the promoter/terminator regions were used in the *fcpB* constructs, even a short truncation of these especially in the promoter would eliminate expression of the *bleo*R gene, whereas much longer sequences were used for both regulatory regions from the *lsu4e* gene. Indeed, almost the entire *fcpB* promoter was missing for one transformant (SM2, Fig. 5), with the *bleo*^R^ gene being expressed instead by the native promoter from the disrupted gene; the construct thereby acting as a “promoter-trap”.

### Gene mutations and segregation

In most of the mapped transformants, the DNA construct had recombined in or closely adjacent to recognizable gene models within the *S*. *marinoi* genome (Fig. 5). Many of these mapped genes encoded hypothetical proteins or proteins of unknown function, whereas the others had low to moderate sequence similarity (30–60%) to known proteins in other diatoms. Given that the initial integration of the DNA construct into each gene region would have occurred in a single allele, similar to when the T-DNA first inserts into the plant nuclear genome also by nonhomologous recombination, we next examined if any of the gene mutations had segregated to homogeneity. This was especially important given that the prevalence of different developmental stages and rates of meiotic division during sexual reproduction of chain-forming diatoms such as *S*. *marinoi* remain uncertain^35^. Using DNA extracted from each transformant, PCR amplification of the disrupted genomic region was performed using oligonucleotides that anneal 250–400 bp either downstream or upstream of the insertion site. As shown in Fig. 5, six of the transformants (SM1, SM7, SM11-12 and SM15-16) were clearly heterozygous, with PCR products derived from both the wild type allele and that carrying the insertional disruption. It was equally clear that the other four transformants (SM2, SM4, SM10 and SM13) were homozygous for the gene mutation, with no PCR product corresponding to the wild type allele. It should be noted that the identity of all PCR products was verified by DNA sequencing, with variations in length of the PCR product corresponding to the mutant allele due to the degree of truncation at the ends of the inserted DNA construct. It should also be mentioned that in two of the transformants (SM4 and SM12), the mutant allele was not observed after the PCR amplification, despite the genomic insertion site for both being confirmed at each end of the construct. More extensive sequencing at each end of the construct revealed that in both transformants a tandem of at least two copies of the construct had inserted at the mapped genomic region. In the case of SM4, a length of fragmented genomic DNA was also found at one end, suggesting that the genomic insertion event in SM4 and SM12 consisted of a tandem of multiple DNA constructs fused together with or without the addition of fragmented *S*. *marinoi* genomic DNA. The long length of this inserted DNA would therefore be difficult to PCR amplify using oligonucleotides specific for the genomic sites each side of the insertion, which could explain the lack of an observable mutant allele for both SM4 and SM12 (Fig. 5). The occurrence of such long tandem/chimeric arrangements of a transformed DNA construct inserted into the genome has again been previously observed for the green algae *C*. *reinhardti*^34^.

## Discussion

Diatoms constitute one of the most ecologically successful groups of marine phytoplankton, responsible for almost a quarter of global photosynthetic carbon fixation as well as the geochemical cycling of various nutrients and minerals. Despite their importance, much remains unknown about the specific functions of most diatom proteins within the different metabolic, biosynthetic and regulatory pathways. One complicating factor for this is the complex evolutionary history of diatom genomes, as revealed in recent years by whole-genome sequencing for several different species. We have recently added to this knowledge with the sequenced genome of the centric diatom *S*. *marinoi*, the first for a true chain-forming species. *S*. *marinoi* is one of the more abundant species within the temperate coastal regions in which diatoms dominate the phytoplankton communities. Its genome is ca. 55 Mb and contains an estimated 22,440 genes, of which 79% encode proteins of unknown function.

One of the most informative genetic approaches for determining specific gene functions underlying different biological traits is mutagenesis, disrupting gene integrity and characterizing the resulting phenotypes. Mutagenesis studies for diatoms have so far been limited to single genes within few species, of which the pennate *P*. *tricornutum* is the most commonly used. More extensive mutagenesis programs have yet to be established for a diatom, although genetic transformation of several different species has been shown over the last 20 years^18–24^. Despite the success of such transformations, none of these studies to our knowledge identified the insertion site of the introduced exogenous DNA within the nuclear genome. To address this issue, we developed a new TAIL-PCR method that reliably identified the genomic insertion site within each *S*. *marinoi* transformant tested, with the only exceptions being those in which the insertion occurred within repetitive regions of the genome, or when a genomic DNA fragment presumably ligated to one end of the construct prior to insertion. The mapped transformants also revealed a high proportion of insertions within recognizable gene models. This suggests that nonhomologous recombination of exogenous linear DNA within diatom genomes, although theoretically random instead favors more accessible regions of high gene expression, a phenomenon also demonstrated for T-DNA insertions in the vascular plant *A*. *thaliana*^36^. It should be noted that although TAIL-PCR did prove to be very reliable, it was relatively time consuming and expensive, which could become an increasingly important consideration for future large-scale mapping of transformants.

An added challenge for mutagenesis studies of a chain-forming diatom such as *S*. *marinoi* was the uncertainty over segregation of the introduced construct and the ability to eventually produce homozygous mutants. Reducing chain lengths within cultures prior to electroporation by vigorous shaking helped minimize the number of non-transformed cells in each chain. Subsequent reculturing of transformants under selection also revealed a relatively high proportion of auxospore-like cells within each culture, suggesting that sexual reproduction and meiotic division in *S*. *marinoi* is triggered by the presence of zeocin. This is likely why nearly half of the mapped transformants were already homozygous for the introduced construct after only several weeks of reculturing. Interestingly, continued monitoring of the heterozygous transformants has yet to reveal further segregation to homogeneity, suggesting that complete inactivation of the disrupted genes in these lines could be lethal.

Given the large proportion of hypothetical proteins within diatom genomes, mutagenesis will increasingly become an important tool for elucidating gene function. With the molecular tools now in place for identifying specific mutants, we believe *S*. *marinoi* will become an invaluable addition to the genetic model species for marine diatoms. As a model species, *S*. *marinoi* has a number of additional advantages, especially from ecological and physiological viewpoints. It is a diatom that dominates phytoplankton assemblages in global temperate coastal regions, with the strain used in this study having only recently been isolated from experimental field sites in Scandinavian waters. The strain is also easy to isolate and grows well under laboratory culturing conditions. *S*. *marinoi* represents centric diatoms that form extensive chains, with fully-silicated frustules. Although present throughout the year, its populations rapidly increase during spring blooms, while also forming benthic resting stages that eventually sediment to the ocean floor^4^. These resting stages can survive in anoxic sediments for over a hundred years and can be revived from different time periods for micro-evolutionary studies on genetic and phenotypic changes^4,5^. As a consequence, *S*. *marinoi* will become an invaluable addition to the functional genomic studies on marine diatoms that have so far been mostly focused on *P*. *tricornutum* and *T*. *pseudonana*.

## Supplementary information

dataset 1
