# Supplementary material for: Skeletonema marinoi as a new genetic model for marine chain-forming diatoms
Source: Sci Rep. 2019 Apr 2;9:5391. doi: 10.1038/s41598-019-41085-5 (PMC6445071; doi:10.1038/s41598-019-41085-5)
Supplement: Supplementary file 1 — dataset 1 [file 41598_2019_41085_MOESM1_ESM.pdf]

Skeletonema marinoi: A new genetic model for marine chain-forming diatoms

Oskar N. Johansson[1], Mats Töpel[2,3], Matthew I.M. Pinder[2], Olga Kourtchenko[2], Anders Blomberg[4], Anna Godhe[2], and Adrian K. Clarke[1]

[1]Department of Biological and Environmental Sciences, University of Gothenburg, Box 461, SE-40530 Gothenburg, Sweden.

[2]Department of Marine Sciences, University of Gothenburg, Box 462, SE-40530 Gothenburg, Sweden.

[3]Gothenburg Global Biodiversity Center (GGBC), Box 461, SE-40530 Gothenburg, Sweden.

[4]Department of Chemistry and Molecular Biology, University of Gothenburg, Box 462, SE-40530 Gothenburg, Sweden

Correspondence should be addressed to A.K.C. (adrian.clarke@bioenv.gu.se).

.

fcpB/bleo ^{R}

agtcggacataccttcagcgtcgtcttcactgtcacagtcaactgacagtaatcgttgatccggagagattcaaa
attcaatctgtttggacctggataagacacaagagcgacatcctgacatgaacgccgtaaacagcaaatcctggt
tgaacacgtatccttttggggcctccgctacgacgctcgctccagctggggcttccttactatacacagcgcgc
atatttcacggttgccagatgtcaagatggcaaagttgacaagtgctgttccagtgctcacagcacgtgatgttg
caggagcagttgagttctggacagatcgtctcggattctcacgagattttgtggaggatgatttcgcaggtgtgg
tccgtgatgatgtgacacttttcatcagtgcagtccaagatcaagtggtgccagataatactcttgcatgggtgt
gggtgcgtggacttgatgagctctacgcagaatggtctgaagtagtgtctacaaatttccgtgatgcatcaggac
cagcaatgacagagattggagaacaaccatggggacgtgaatttgcacttcgtgatccagctggaaattgcgtgc
atttcgtggcagaagagcaagattaaaccttccttaaaaatttaattttcattagttgcagtcactccgctttgg
tttcacagtcaggaataacactagctcgtcttcaccatggatgccaatctcgcctattcatggtgtataaaagtt
caacatccaaagctagaacttttggaaagagaaagaatatccgaatagggcacggcgtgccgtattgttggagtg
gactagcagaaagtgaggaaggcacaggatgagttttctcgagtccgact

lsu4e/bleo ^{R}

agtcggatcgactcaagctcctttgccctgacagacggaagcggtggaagtgcagctgcagccatatct
gctgttcactttccctgaatctgatctgtcctctctctagaggattacctactgtgctctcagttgtt
gcttgagacccaccacacgagaatgatttcatggagttttggagatttcgagatcttcggtattttct
tcgtacaatccacttttcccatcggacatgaccggacacaatttggcctggattctgtagatagacaa
catgtaagtacccacaaatacattacttctaattgatatttctgtacatacattgtacagaggaatcc
gtaaaactctccataccaatttgagtaaatggtaaaggcgcaaacctctcctccgccaagaaactcac
cactcccaactcgaccttttcaaccctgttgcagcggtgattacaattcgtcaactgaccggaccgca
ccttaggaacgaaggtattaaacaacaatcaaaatggcaaagttgacaagtgctgttccagtgctcacagca
cgtgatgttgcaggagcagttgagttctggacagatcgtctcggattctcacgagattttgtggaggatgatttc
gcaggtgtggtccgtgatgatgtgacacttttcatcagtgcagtccaagatcaagtggtgccagataatactctt
gcatgggtgtgggtgcgtggacttgatgagctctacgcagaatggtctgaagtagtgtctacaaatttccgtgat
gcatcaggaccagcaatgacagagattggagaacaaccatggggacgtgaatttgcacttcgtgatccagctgga
aattgcgtgcatttcgtggcagaagagcaagattaagtcaaatagtaaaaataaagaaaatcgataaacagtgaa
acggcacagtccactcttcttacatatctatctctcatattacgatatcactgatccaactttacagtgacggtt
gatcagaagtcagatccatttttttgtttgccagctcaaatgcctttaattacctgagaagcctaccgtgcccaca
aacaactgtaggtacttcaaatgcatgtacacatttgattctttctattgtttgcttctctttcataacattcat
tagcatgagaataatcatacacatcaagatgacgttctaaattatttattactctatatcaatcaacaactgtat
gaaattacttatttctcagaaatctcttcgaagttgccatcaacaaggtctggtacatcatcatcgtcgtcatcc
tcttcaactccctttttgaacatctcctcccataccctgggcagccattgcttgctgcaattgtgcaatgttatcc
atgccaagttgtccgact

Supplementary Figure 1. Nucleotide sequences of the two linear DNA constructs used to transform *S. marinoi*. The promoter and terminator regions for both the *fcpB* gene from *P. tricornutum* and the *lsu4e* gene from *S. marinoi* are shown in red and blue, respectively, with the gene conferring resistance to the antibiotics zeocin/bleomycin (*bleo*^{R}) shown in black.

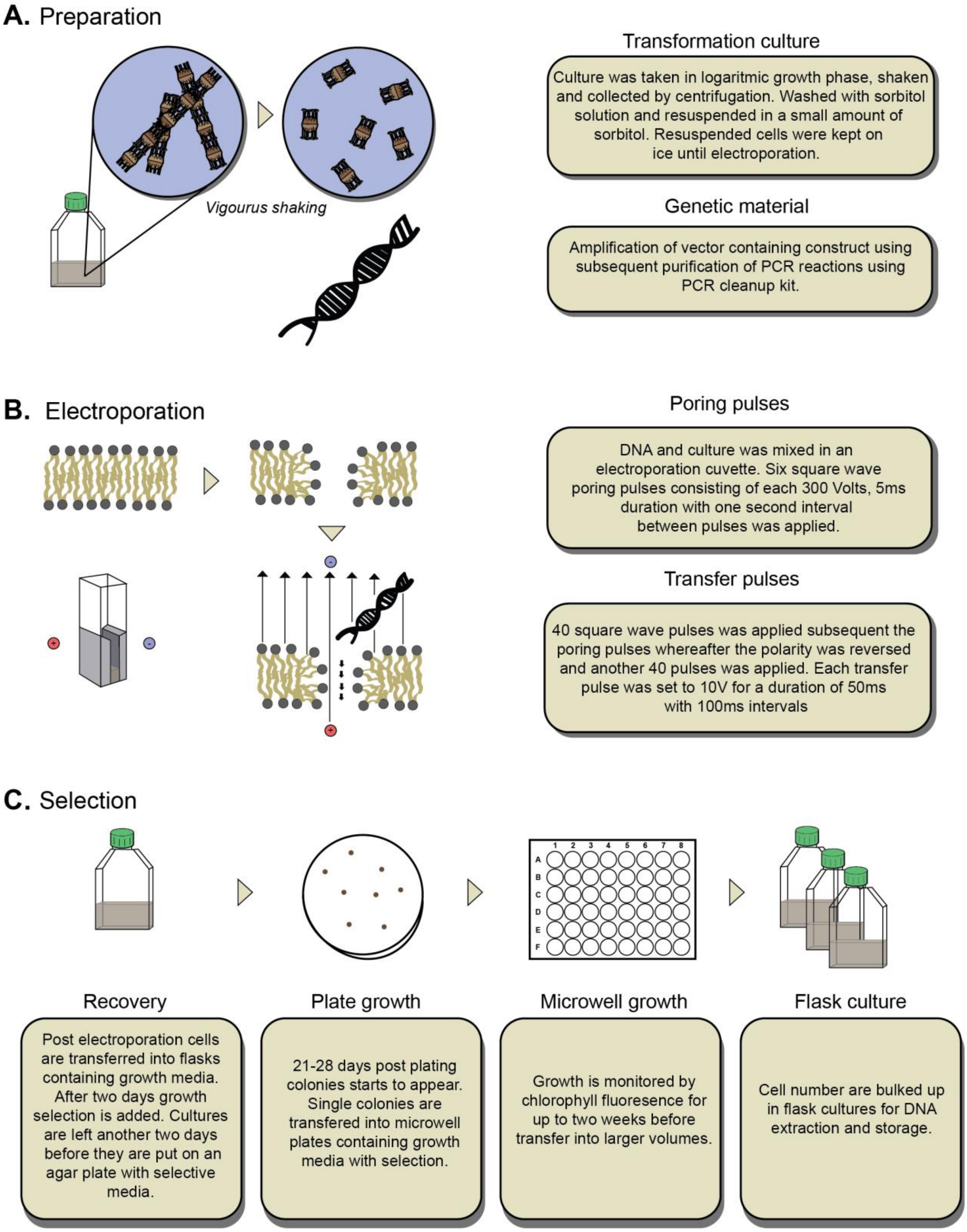

Supplementary Figure 2. Overview of the transformation procedure for *S. marinoi*. **A.** Preparation of single cell cultures and purification of linear DNA fragments. **B.** Electroporation. **C.** Post transformation selection and monitoring

Supplementary Figure 3. Electroporation Settings

Setting	Range
**Poring pulses**	
Voltage	300-500 V
Pulse Interval	0.1-5 s
Pulse Length	0.1-20 ms
Number of pulses	2-10
**Transfer pulses**	
Voltage	10V
Pulse Interval	0.1 s
Pulse Length	50 ms
Number of pulses	40 + 40 (shift of polarity in between)
**General**	
DNA range	2-10 µg/cuvette
Cell concentration	5×10^7 – 2×10^8 cells ml^{-1}
Osmolarity	0.3-0.5 M sorbitol
Temperature	4°C

Supplementary Figure 4. List of primers

Primer	Sequence (5'->3')
**Transformation**	
fcpB-forward	AGTCGGACATACCTTCAGCG
fcpB-reverse	AGTCGGACTCGAGAAAACTC
Lsu4e-forward	AGTCGGATCGACTCAAGCTCC
Lsu4e-reverse	AGTCGGACAACTTGGCATGG
**TAIL-PCR**	
SAD1	**ACGATGGACTCCAGAG**VNVNNNGGAA
SAD2	**ACGATGGACTCCAGAG**BNBNNNGGTT
SAD3	**ACGATGGACTCCAGAG**VVNVNNNCCAA
SAD4	**ACGATGGACTCCAGAG**BDNBNNNCGGT
SAD5	**ACGATGGACTCCAGAG**VNVNNNGGAA**C**
SAD6	**ACGATGGACTCCAGAG**BNBNNNGGTT**C**
SAD7	**ACGATGGACTCCAGAG**VVNVNNNCCAA**G**
SAD8	**ACGATGGACTCCAGAG**BDNBNNNCGGT**C**
SAD9	**ACGATGGACTCCAGAG**VNVNNNGGAA**CC**
SAD10	**ACGATGGACTCCAGAG**BNBNNNGGTT**CC**
SAD11	**ACGATGGACTCCAGAG**VVNVNNNCCAA**GG**
SAD12	**ACGATGGACTCCAGAG**BDNBNNNCGGT**CC**
SSMP	**ACGATGGACTCCAGAG**
BLEO1	GCAAAGTTGACAAGTGCTGTTCCAG
BLEO2	CTTGCTCTTCTGCCACGAAATGC
BLEO3	AGCACTGGAACAGCACTTGTCAACTTTGC
BLEO4	GCGTGCATTTCGTGGCAGAAGAGCAAG
BLEO5	GAGAATCCGAGACGATCTGTCCAGAACTC
BLEO6	CCTGCGAAATCATCCTCCACAAAATCTCG
BLEO7	GTGAATTTGCACTTCGTGATCCAGCTGG
BLEO8	GCATCAGGACCAGCAATGACAGAGATTGG
**Confirmation**	
SM1a	ACCGTCAAATTAGCAGCCCGTGATGG
SM1b	GGACACATAACTCATGCCATCGTCGC
SM2a	ACAGAGAGAGGGTCAACAAGATAATGG
SM2b	CGAGCGCCGTGCAAAAGGAAG
SM4a	ATTCTCCTCGTTCTTCTGTTGAACATC
SM4b	CCTTTTGAGGCGATGATGCAATAAC
SM7a	GATTCTACGACGACGGCGACAACTAC
SM7b	GATCCAGGTTTAGGACGTTGGCCAG
SM10a	CCCTTCATTAAGTACAGACAGTGACG
SM10b	CTCAAGATCGAAAGGACGATAGTAGG
SM11a	GGAAGTGATTGATGCTCCTGAGAAGG
SM11b	GTGAAATGGCACCCAAGGACACCA
SM12a	GATGAAGTGAGGCAGAGGCCACTTG
SM12b	CAGAGAGCACGGCTGCAGCTCAGCG
SM13a	AACGTGCGTACTACCAACAATGAGTAC
SM13b	GTTACCACCAAACAACCCGTCGC
SM15a	CGAACCTCATTTATCACCCTACTAGAC
SM15b	CAGAACGTGTTAAGATTTTGCAGGAAGC
SM16a	CTCTTGCTCTCCTTTGACAGATGTAACCTTA
SM16b	GCTTGGTTGTGGATGGCGCAAAG

Supplementary Figure 5. Thermal conditions for the modified TAIL-PCR approach used to map the genomic insertion sites in *S. marinoi* transformants.

Primary reaction (1°)			Secondary reaction (2°)			Tertiary reaction (3°)		
**Step**	**Temp.**	**Time**	**Step**	**Temp.**	**Time**	**Step**	**Temp.**	**Time**
1	95°C	5 min	1	94°C	20 s	1	94°C	20 s
2	95°C	1 min	2	65°C	1 min	2	68°C	1 min
3	94°C	30 s	3	72°C	3 min	3	72°C	3 min
4	60°C	1 min	4	Go to 1	1 x	4	94°C	20 s
5	72°C	3 min	5	94°C	20 s	5	68°C	1 min
6	Go to 3	10 x	6	68°C	1 min	6	72°C	3 min
7	94°C	30 s	7	72°C	3 min	7	94°C	20 s
8	25°C	2 min	8	94°C	20 s	8	50°C	1 min
9	72°C ramp 0.5°C / sec	3 min	9	68°C	1 min	9	72°C	3 min
10	72°C	3 min	10	72°C	3 min	10	Go to 1	7 x
11	94°C	20 s	11	94°C	20 s	11	72°C	5 min
12	58°C	1 min	12	50°C	1 min	12	4°C	∞
13	72°C	3 min	13	72°C	3 min			
14	Go to 11	25x	14	Go to 5	13x			
15	72°C	5 min	15	72°C	5 min			
16	4°C	∞	16	4°C	∞			

Supplementary Figure 6. Details of the mapped DNA construct integrated into the genome of selected *S. marinoi* transformants. Shown is the transformant line, the type of transformed DNA construct, the relative genomic insertion site within recognizable gene models or intergenic regions, whether the genotype of the insertion is homozygous or heterozygous, and the closest ortholog to the disrupted gene model if available in GenBank.

Line	Promoter	Insertion	Genotype	Match in NCBI GenBank	Match Sequence ID
SM1	fcpB	5'-UTR	Heterozygous	*T.pseudonana* CCMP1335 predicted protein, mRNA)	XM_002288967.1
SM2	fcpB	1st Exon	Homozygous	*P. tricornutum* CCAP1055/1 G-protein coupled GABA receptor	XM_002176403.1
SM3	fcpB	Intergenic	n.d.	-	-
SM4	lsu4e	5'-UTR	Homozygous	No match in GenBank	-
SM5	lsu4e	MGF	n.d.	*Fragilariopsis cylindrus* CCMP1102 hypothetical protein	OEU16049.1
SM6	lsu4e	Intergenic	n.d.	-	-
SM7	lsu4e	1st Exon	Heterozygous	No match in GenBank	-
SM8	lsu4e	Intergenic	n.d.	-	-
SM9	lsu4e	Intergenic	n.d.	-	-
SM10	lsu4e	3rd Exon	Homozygous	*T. pseudonana* CCMP1335 predicted protein	XP_002296510.1
SM11	lsu4e	1st Exon	Heterozygous	*T. pseudonana* CCMP1335 predicted protein, mRNA	XM_002292138.1
SM12	lsu4e	1st Exon	Heterozygous	No match in GenBank	-
SM13	lsu4e	1st Exon	Homozygous	*T. pseudonana* CCMP1335 predicted protein, mRNA	XM_002296474.1
SM14	lsu4e	MGF	n.d.	*T. oceanica* hypothetical protein THAOC_08313	EJK70335.1
SM15	lsu4e	2nd Exon	Heterozygous	*T. pseudonana* CCMP1335 GMP synthase	XM_002289176.1
SM16	lsu4e	5'-UTR	Heterozygous	*T. pseudonana* CCMP1335 predicted protein	XM_002286015.1

n.d., not described; MGF, multigene family
